# Human Systems Immunology in the Omics Era: Challenges, Methods, and Emerging Directions

**DOI:** 10.1002/eji.70164

**Published:** 2026-03-13

**Authors:** Lennart Riemann, Reinhold Förster

**Affiliations:** ^1^ Hannover Medical School Institute of Immunology Hannover Germany; ^2^ Department of Paediatric Pneumology Hannover Medical School Allergology, and Neonatology Hannover Germany; ^3^ Excellence Cluster RESIST (EXC 2155) Hannover Medical School Hannover Germany

**Keywords:** computational immunology, high‐dimensional data, human cohort studies, multi‐omics integration, systems immunology

## Abstract

The human immune system is a highly complex, dynamic, and heterogeneous network shaped by genetic, environmental, and temporal influences. Advances in high‐throughput omics technologies have transformed our ability to study this complexity directly and comprehensively in human cohorts. These developments have positioned systems immunology as a powerful framework for investigating coordinated immune responses, identifying regulatory mechanisms, and linking molecular patterns to clinical phenotypes. However, the analytical challenges inherent to large‐scale, multimodal datasets—including batch effects, small sample sizes, high dimensionality, and substantial interindividual heterogeneity—require rigorous study design, robust statistical modeling, and thoughtful data analysis strategies. In this review, we summarize key technological foundations enabling modern human systems immunology, outline common analytical pitfalls and effective mitigation approaches, discuss data integration concepts, and highlight emerging opportunities in the field. Together, these technological and analytical advances are redefining how immune function is measured and interpreted in real‐world human biology and hold significant promise for enhancing mechanistic insight, biomarker discovery, and precision medicine across immunological diseases and interventions.

## Graphical abstract

Systems immunology provides a transformative framework for decoding human immune complexity. This review evaluates high‐throughput technologies and provides a roadmap for navigating analytical challenges—such as batch effects and interindividual heterogeneity—to successfully translate multimodal data into meaningful biological insights.

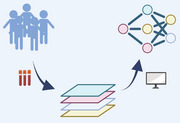

## 1 Introduction

The human immune system is a complex and dynamic defense network that is composed of hundreds of distinct cell types as well as a plethora of effector and signaling molecules [1]. Understanding how this intricate system functions in health and disease is a central goal of immunological research. Immune dysregulation underlies a wide spectrum of human conditions—including autoimmune diseases, allergies, and cancer—and is increasingly recognized as having a significant role in aging and chronic noncommunicable diseases [2, 3, 4, 5, 6, 7, 8, 9, 10]. Progress in this area holds promise for enhancing prevention, diagnosis, and treatment across clinical disciplines.

Yet, unraveling immune responses in different contexts remains an extraordinary challenge due to the vast number of immune parameters as well as extensive immune variability across individuals, within the same individual over time, and across populations [11, 12, 13, 14, 15, 16, 17, 18, 19, 20, 21, 22].

To meet this challenge, a growing number of studies are now leveraging systems immunology approaches to gain holistic insights into immune function, regulation, and variation. This review highlights recent conceptual and technical advances in human systems immunology, outlines key analytical challenges and potential strategies to address them, and discusses emerging opportunities for future research.

### 1.1 Capturing Immune Complexity in the Real World

**FIGURE 1:**
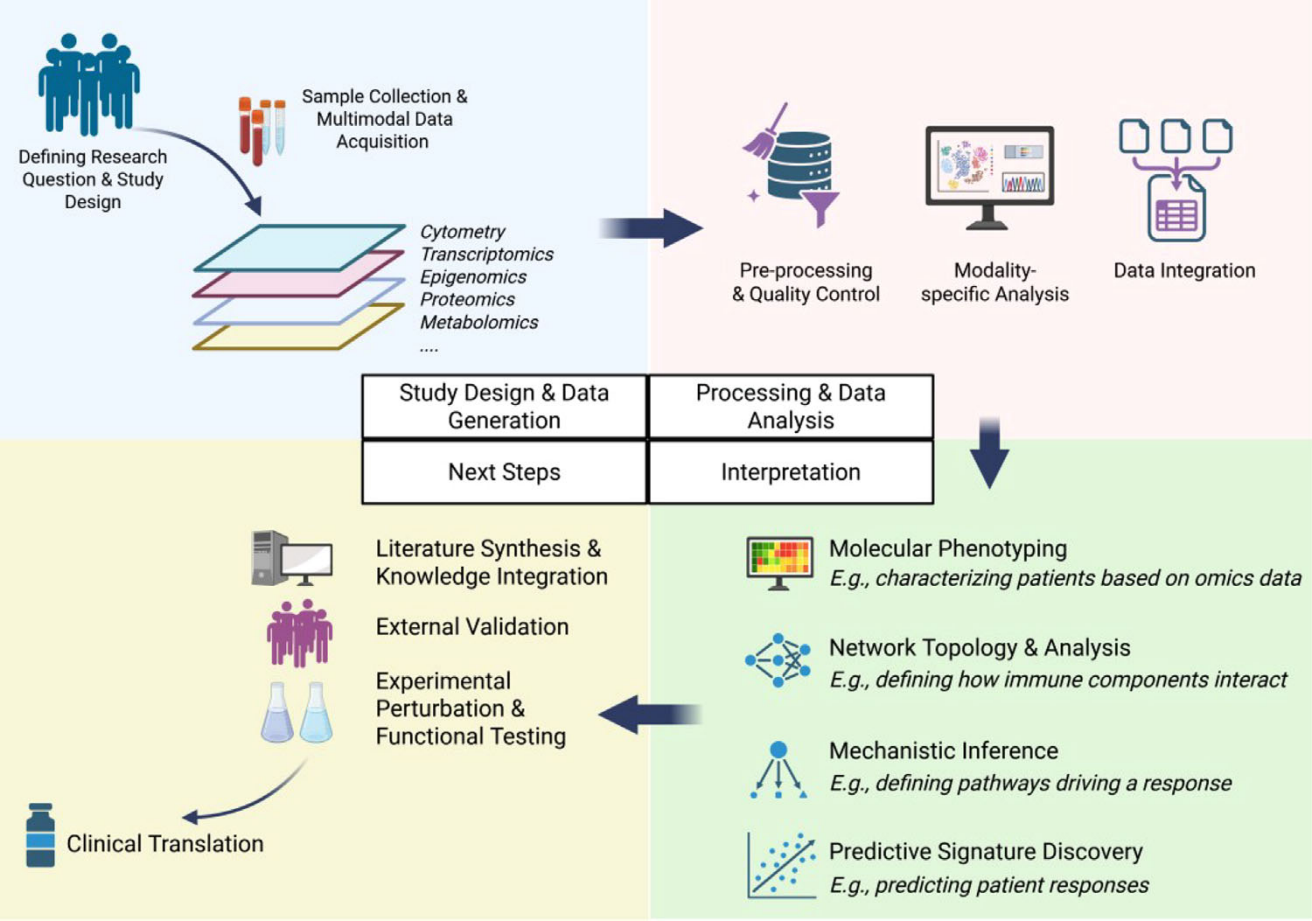
Overview of the human systems immunology analytical workflow. **Top‐left: Study Design and generation**. The process begins with a clearly defined research question and a robust study design (e.g., considerations of cohort selection, sample size, and sampling strategy) tailored to the clinical or biological objective. Multimodal data acquisition (e.g., transcriptomics, proteomics, cytometry, and other omics layers) is performed on human biospecimens using standardized protocols. **Top‐right panel: Data processing and analysis**. For each modality, rigorous preprocessing and quality control (QC) are performed to ensure data integrity and to address technical challenges such as batch effects or missing data. Modality‐specific and integrative computational frameworks are then applied to analyze individual datasets, followed by data integration to achieve a holistic view of the immune system and to uncover relationships across molecular layers. **Bottom‐right: Biological interpretation**. Analytical results are interpreted in the context of the study's scientific aims, such as high‐resolution immune phenotyping of patients, inference of cellular interactions or regulatory networks, elucidation of underlying immunological mechanisms, or identification of predictive molecular signatures associated with clinical outcomes. **Bottom‐left: Validation and translation**. Computational findings are contextualized using existing literature and prior knowledge to assess biological plausibility. Where possible, results are validated in independent cohorts and tested experimentally (e.g., through in vitro assays). Ultimately, validated discoveries may support clinical translation, for example, by enabling patient stratification or informing the development of immune‐targeted therapeutic strategies. Figure created with BioRender.

Systems immunology is an interdisciplinary field that integrates high‐throughput experimental technologies with advanced computational and statistical approaches to achieve a holistic, quantitative understanding of immune responses. By interrogating multiple layers of the immune system, these studies aim to characterize the coordinated behavior of immune components, uncover mechanisms of immune regulation, and improve prediction of disease trajectories and treatment outcomes (Figure 1). These approaches have already significantly advanced our understanding in areas such as human immune system development, vaccinology, autoimmune disease, and cancer immunotherapy [23]. For example, a landmark systems immunology study by Olin et al. mapped the development of the neonatal immune system across the first months of life, revealing a stereotypic developmental program that is shared by preterm and term children [24]. Applying similar systems approaches to vaccinology, recent work identified a platelet‐associated transcriptional signature that accurately predicted the long‐term durability of antibody responses across multiple vaccines, thus uncovering a predictive biomarker with potential relevance for next‐generation vaccine design [25].

While preclinical models remain indispensable for mechanistic discovery, their translational limitations have also become apparent [23, 26]. For example, many interventions that demonstrate efficacy in animal models fail to show clinical benefit in humans, underscoring species‐specific differences in immune responses, disease mechanisms, and physiological context. In particular, commonly used inbred mouse strains lack the genetic diversity and environmental exposures that shape human immune function, limiting the generalizability of findings derived from these controlled systems [26]. By contrast, systems immunology studies can capture the full complexity of human biology and are therefore uniquely positioned to yield insights that are more immediately relevant to clinical translation. For instance, in one multi‐omics study of 442 primary bone marrow samples from children and adults with acute myeloid leukemia, the assessment of transcriptional and proteomic profiles revealed specific interferon‐γ signatures that predicted chemotherapy resistance and responsiveness to immunotherapy [27]. Such systems‐derived findings move beyond single biomarkers and may enable more precise patient stratification to select the optimal therapies for each individual [28, 29].

The wealth of insights that can now be drawn from human cohort studies has been enabled by the emergence of high‐throughput and high‐bandwidth technologies that generate datasets capturing molecular and cellular information at unprecedented resolution and scale.

### 1.2 Technological Foundations of Systems Immunology

Modern experimental technologies have fundamentally transformed the study of immune responses and laid the groundwork for systems‐level analyses. The number of measurable features per sample across various biological layers has expanded dramatically. Whereas early immunological studies were limited to analyzing one or a few parameters per sample, contemporary high‐throughput platforms now enable the simultaneous interrogation of hundreds to thousands of cellular and molecular features. These advances are exemplified by developments in cytometry (e.g., spectral flow cytometry and mass cytometry [CyTOF]), multiplex proteomics, and next‐generation sequencing.

Single‐cell RNA sequencing (scRNA‐seq) has transformed immune profiling by enabling the measurement of transcriptomes in thousands to millions of individual cells, shifting the resolution from bulk averages to single‐cell granularity. Multiple platforms exist, including droplet‐based and plate‐based protocols, which differ in throughput, sensitivity, and sequence coverage, and should be chosen based on the specific research question [30]. ScRNA‐seq is particularly powerful for revealing cellular heterogeneity, including identifying rare cell populations, uncovering diverse activation states, and profiling developmental or differentiation trajectories of cells. For example, a recent study dissected the cellular heterogeneity of non‐small‐cell lung cancer in response to immunotherapy, identifying particular precursor exhausted T cells that correlated with patient survival [31].

Beyond transcriptomics, single‐cell technologies can be combined with assays of chromatin accessibility (e.g., scATAC‐seq [32]), T and B cell receptor repertoires [33], and surface protein expression (e.g., CITE‐seq [34]). Multimodal single‐cell profiling integrates these within the same cell, allowing simultaneous investigation of regulatory programs, phenotypic states, protein expression, and antigen receptor diversity [35]. Using multi‐omics single‐cell approaches, researchers have, for instance, profiled the human immune response to SARS‐CoV‐2 infection at unprecedented depth, identifying specific cellular subsets and states associated with disease severity [36]. Cancer immunology is another area where single‐cell multi‐omics studies led to many novel insights [37, 38], uncovering, for example, transcriptional regulators of cellular heterogeneity in glioblastoma [39]. Several large‐scale repositories now provide multimodal data collected from the same cell, such as the Cancer Genome Atlas (TCGA) [40] and the COvid‐19 Multi‐omics Blood Atlas (COMBATdb) [41], providing valuable resources for integrative systems analyses.

Spatial omics technologies add an additional dimension to molecular profiling by providing information on the spatial localization of cells and molecules within tissues. Spatial transcriptomics allows mapping of transcripts within tissues, ranging from single‐cell to multicellular spot resolution, using sequencing‐ or imaging‐based approaches that differ in spatial precision, transcript coverage, and throughput [42, 43]. These methods enable the analysis of the location of cell populations in tissues, the identification of spatially variable genes, and the characterization of tissue microenvironments [42]. Spatial transcriptomics has, for instance, been used to map and refine the architecture and microenvironment of healthy tissues, such as the human cortex [44], as well as those of pathological tissues, including cancers [45] and chronic diseases such as pulmonary fibrosis [46].

Spatial transcriptomics can be combined with other assays, including spatial proteomics or epigenomics, to include complementary molecular readouts [42, 43]. For example, simultaneous analysis of spatial proteomics and transcriptomics can improve the interpretation of cell types, since correlations between transcript and protein abundance are often limited, reveal receptor–ligand interactions, and link transcriptional cell states to functional protein expression in situ [42, 47]. One study, for instance, integrated gene and protein expression to profile and spatially resolve immune responses in the human renal papilla in patients with kidney stone disease, identifying specific matrix metalloproteinases associated with active stone formation [48]. In the future, spatial omics data may also be used in clinical contexts, for example, to predict treatment responses in cancers, and developments toward scalable, clinically compatible platforms are already underway [43, 49].

Omics datasets can be further complemented by additional orthogonal datasets capturing demographic and clinical metadata, laboratory values, or imaging results. Together, these multimodal datasets provide a comprehensive and multidimensional view of the immune system that is more informative than any single modality alone. One recent study, for instance, integrated five omics layers with detailed clinical metadata in people living with HIV to generate a multilayer atlas of immune variability and comorbidity patterns in this population, highlighting distinct molecular pathways of systemic inflammation and underlying comorbidities [50]. In cancer immunology, Petralia et al. [51] recently analyzed over 1000 tumor samples to define conserved immune subtypes across 10 cancer entities and characterized genomic, epigenetic, transcriptomic, and proteomic changes associated with each, revealing specific kinase activities in immune subtypes that may be potential targets for immunotherapy. In another study [52], researchers integrated single‐cell transcriptomics to define 12 cross‐tissue cellular module networks and mapped them onto spatial data, enhancing our understanding of how tissue configurations change dynamically across age and how healthy tissue organization is lost during cancer progression.

These examples demonstrate how the depth and breadth of these omics datasets, particularly in combination, offer unprecedented opportunities to uncover mechanistic and translationally relevant insights. Yet their scale and complexity also introduce significant analytical challenges, requiring the development and application of novel statistical approaches to ensure valid and biologically meaningful results.

### 1.3 Key Analytical Considerations

These challenges span multiple domains across the full data lifecycle, from data preprocessing and quality control to statistical modeling and biological interpretation. Addressing these challenges requires careful methodological planning and domain‐specific expertise. Key considerations, along with potential mitigation strategies, are summarized in Table 1 and outlined below.

**TABLE 1:** Overview of key data analysis challenges and example mitigation strategies in human immunology studies.

Batch effects	Sound experimental design (e.g., randomization of samples across batches) Batch correction methods (e.g., ComBat‐seq for bulk‐RNAseq data) Statistical models with adjustment for batches/plates
Missing data	Filtering sparse features Single/multiple imputation
Noise and low‐abundance features	Filtering of low‐expression features (e.g., transcripts) Use of shrinkage estimators (e.g., empirical Bayes moderation in limma)
High dimensionality, low samples size (HDLSS datasets)	Dimensionality reduction (e.g., PCA, CytoMod) Multiple testing correction Regularization techniques during statistical modeling (e.g., LASSO regression)
Pattern discovery in unlabeled high‐dimensional data	Unsupervised clustering (e.g., FlowSOM) Network‐based approaches (e.g., WGCNA)
Interindividual variation	Covariate adjustment in regression models Mixed‐effect models Stratified analyses
Longitudinal data	Time‐series modeling approaches Trajectory inference
Multimodal data integration	Correlation networks Multi‐omics integration approaches, including machine learning frameworks

Technical variation and batch effects are important factors requiring attention during data preprocessing. Human studies often require large sample sizes—often in the hundreds—to overcome interindividual variability and ensure sufficient statistical power. Accordingly, processing such sample numbers typically requires batch‐wise experimental handling, which introduces technical variation (i.e., due to changes in reagent lots or instrumentation drifts) [53]. These sources of unwanted variation can confound biological signals across diverse data modalities (e.g., cytometry, transcriptomics, and proteomics) and therefore require careful assessment and mitigation [53, 54].

Mitigation begins at the design stage. Rigorous experimental planning—such as random distribution of samples across batches, inclusion of reference controls in every batch, and systematic recording of batch metadata—greatly reduces downstream corrective burden. During analysis, visual inspection for batch effects using PCA or UMAP, along with modality‐specific diagnostic plots (e.g., marker density plots for cytometry), provides an initial assessment of batch structure. For single‐cell data, dedicated methods have been developed to assess and quantify batch effects at both gene and cellular levels [55]. When batch effects are detected, a range of method‐specific correction tools is often available. Examples include CytoNorm [56] and CyCombine [57] for flow cytometry data, ComBat‐Seq [58] and RUVSeq [59] for bulk RNA sequencing data, and Harmony [60], Seurat [61], or scVI [62] for single‐cell data. Regardless of the method, it is crucial to re‐inspect PCA plots and clustering structure before and after correction to ensure that unwanted variation was reduced without distorting the true biological signal. Importantly, batch correction is not appropriate in all situations. If the batch is strongly confounded with the outcome of interest, correction may remove meaningful biology or create artificial structure. In such cases, alternative strategies such as including batch as a covariate in statistical models might be an option. Similar caution applies when integrating datasets from different sources, which is frequently performed in scRNA‐seq studies to increase cell/sample numbers or compare findings across different conditions. A large number of dataset integration techniques have been developed to remove unwanted technical variation in single‐cell data before joint downstream analyses (reviewed in [63, 64]).

Missing data is another frequent issue in multi‐omics studies, for example, due to assay failures or limited sample material, and may limit statistical power or even introduce bias. A first step is to assess the extent, type and pattern of missingness (e.g., missing completely at random, missing at random, or missing not at random). If data missingness is random and not excessive, imputation techniques can often be effectively applied [65]. Single imputation techniques (e.g., Random Forest or k‐nearest neighbors imputation) provide a practical way to fill missing values, but they may underestimate variability and lead to overly confident downstream analyses. Multiple imputation approaches, which generate several plausible datasets and combine results across them, better account for uncertainty and reduce bias [65]. The choice of imputation method should consider data type as well as downstream analysis, and imputation results should be carefully assessed before continuing with the analysis. In high‐dimensional systems immunology datasets, specialized approaches such as matrix factorization can often effectively deal with missing data [66, 67]. Finally, careful reporting of missing data and sensitivity analyses are essential to ensure that conclusions are robust.

Small sample sizes, often the result of high assay costs or limited access to biospecimens, are a common limitation in systems immunology studies. In such settings, estimates of effect sizes, variances, and correlations can be unstable, increasing the likelihood of both false positives and false negatives. Beyond careful study design with sufficient sample sizes, statistical strategies such as regularization, cross‐validation, and borrowing information across features can help mitigate some of these risks and improve the robustness of inferences. Including validation datasets can substantially increase confidence in findings derived from limited sample sizes. In one study by Odak et al. [68], for instance, cytokine signatures identified in a small discovery cohort of vaccine high‐ and low responders were replicated in a second, independent validation cohort, confirming their robustness and supporting their generalizability. Leveraging publicly deposited datasets can further enhance analytical power or serve as external validation datasets, and become increasingly available. For gene expression data, for example, data repositories such as the Gene Expression Omnibus (GEO) and the European Nucleotide Archive (ENA) can be screened for suitable datasets to complement, expand, or validate own datasets.

High dimensionality and multiple testing represent core statistical challenges in omics research. In typical datasets, the number of measured features—such as genes, proteins, or metabolites—vastly exceeds the number of samples, a scenario referred to as the “p ≫ n problem.” This imbalance can lead to model overfitting, inflated false discovery rates, and reduced generalizability [69]. Strategies such as dimensionality reduction, sparse modeling, and the grouping of co‐regulated features into biologically meaningful modules (e.g., gene modules or cytokine clusters) help reduce the number of comparisons and enhance interpretability. For example, in cytokine profiling, methods like CytoMod first group co‐varying cytokines into interconnected modules before testing for clinical associations, reducing the number of statistical tests while capturing the networked nature of cytokine signaling [70]. For example, Cohen et al. [70] grouped 37 cytokines into 3–6 modules in various cohorts and found in multiple instances modules that were significantly associated with a clinical phenotype even when none of the individual cytokines reached statistical significance after multiple testing correction. This illustrates how module‐based aggregation can uncover robust biological associations that would otherwise be missed. Other statistical approaches, such as penalized regression models (e.g., LASSO or Elastic Net), can also mitigate overfitting in high‐dimensional spaces and may be particularly suitable in situations where the number of features greatly exceeds the number of samples or when predictors are highly correlated (e.g., plasma protein levels) [71]. In all cases, adjustment for multiple testing—such as controlling the false discovery rate using the Benjamini–Hochberg procedure—is usually strongly recommended to avoid spurious findings and maintain statistical rigor [72]. However, it is important to recognize that such analyses remain exploratory: a statistically significant test even after multiplicity adjustment does necessarily indicate a true biological effect, while a nonsignificant result does not rule one out. Findings should be interpreted in the context of prior knowledge, effect sizes, and, whenever possible, validated in independent datasets.

Finding biological patterns in high‐dimensional data presents another common challenge in systems immunology, and data‐driven analysis strategies can offer powerful solutions. An illustrative example of this is the analysis of high‐dimensional flow cytometry data. Manual gating—iteratively plotting combinations of two markers in two‐dimensional plots—becomes increasingly impractical for large staining panels and provides only a limited view of the complex data, potentially missing rare or novel cell populations [73].

Data‐driven approaches (e.g., FlowSOM or Phenograph) address these limitations by assigning cells to cell clusters in an unsupervised manner using full marker profiles, enabling unbiased and reproducible discovery of cell populations [74, 75].

A similar approach is also very commonly performed in single‐cell data, where clustering methods are employed to identify and define cell populations by grouping cells into groups with similar expression characteristics [76, 77].

Beyond the challenges of high‐dimensional feature spaces, human studies introduce additional complexity due to the pronounced interindividual variability, which necessitates sophisticated statistical modeling. Regression models can help address this by adjusting for relevant covariates such as age, sex, or comorbidities, while mixed‐effects models account for participant‐specific random effects. For example, cellular frequencies of many immune cell subsets are strongly influenced by age, sex, and cytomegalovirus infection [17, 78, 79], and adjusting for these factors in cytometry profiling studies can help to disentangle other signals of interest. Longitudinal study designs further increase complexity, requiring models that incorporate intraindividual correlations across repeated measurements, such as linear mixed‐effects models or generalized estimating equations (GEE). In systems immunology, where high‐dimensional measurements are frequently combined with longitudinal sampling, these models are essential for disentangling true biological signals from individual variability, enabling more robust and interpretable inferences. In single‐cell studies, trajectory inference frameworks such as Slingshot or Monocle can be used to capture nonlinear immune trajectories and temporal dynamics [80, 81]. Together, these tools provide robust ways to extract meaningful biological insight from complex, heterogeneous, and temporally resolved human immune data.

### 1.4 Multimodal Data Integration

Despite the richness of data generated by modern technologies, each experimental method captures only a fragment of the immune system's current state and function. Multimodal data integration aims to combine complementary information from diverse sources—such as transcriptomics, proteomics, cytometry, and clinical metadata—into a unified analytical framework. Such integration can improve the accuracy of predictive models, enhance biological interpretability, and reveal cross‐modality relationships that remain undetectable in unimodal analyses.

A wide range of integration strategies and computational tools has been developed (reviewed elsewhere [82, 83, 84]), which can be broadly categorized according to the stage at which data fusion occurs: early, intermediate, or late integration. In early integration, features (typically normalized) from each dataset are concatenated into a single unified dataset before modeling [85]. For example, gene expression values and cytokine concentrations might be combined as input for a machine learning model to classify clinical outcomes. This approach is conceptually straightforward and allows direct modeling of cross‐modal interactions. However, it may suffer from imbalanced contributions if one modality dominates in terms of feature count or variance, and may be sensitive to missing data.

Intermediate integration transforms modalities into latent representations—either separately or jointly—within a shared modeling framework. When representations are learned separately for each modality (e.g., via dimensionality reductions) and subsequently fused, this is sometimes also referred to as mixed integration [85]. In contrast, methods such as multi‐omics factor analysis (MOFA) learn joint representations directly and can be used to identify biological factors that simultaneously explain variance across multiple omics layers [86]. The advantage of intermediate integration strategies lies in their ability to preserve essential information while potentially reducing noise through dimensionality reduction. However, modeling interactions across datasets may be limited when modalities are treated independently.

Late integration constructs independent models for each modality and then combines their outputs (e.g., predicted probabilities or class labels) in a second‐stage model [85]. For example, separate predictive models are trained on flow cytometry and clinical data, and outputs are used as inputs for a logistic regression model to predict the outcome. This approach is often robust to missing data, accommodates imbalanced feature sets, and retains modality‐specific strengths. However, like intermediate integration, it may overlook biological insights that emerge only through direct modeling of intermodality interactions.

The choice of integration strategy depends on the research question, the properties of the data, and computational resources. As multimodal datasets become increasingly common in immunology, thoughtful integration strategies will be essential to fully harness their potential and uncover deeper, cross‐layer insights into immune function.

### 1.5 Emerging Directions and Artificial Intelligence in Systems Immunology

Several emerging developments promise to further expand the scope and impact of systems immunology. One key avenue is the broader integration of biospecimens beyond peripheral blood. While blood remains the most accessible and widely used tissue for immune profiling, incorporating samples such as mucosal swabs, cerebrospinal fluid, or gut microbiota can provide compartment‐specific insights. In addition, the rise of digital health technologies—including wearable sensors and smartphone‐based monitoring—enables real‐time collection of longitudinal physiological and behavioral data (e.g., sleep, physical activity, heart rate variability) [87, 88, 89]. Correlating these parameters with molecular immune profiles opens new possibilities for studying the dynamic interplay between environment, behavior, and immune function. Similarly, integrating exposomic data such as air pollution, diet, or psychosocial stress may help unravel how external factors shape immune trajectories, as exemplified by the HELIX study in early‐life exposome research [90].

These expanding data sources call for study designs that can integrate and interpret them effectively. Clinical trials represent an excellent opportunity to embed systems‐level analyses in controlled, prospective human studies. They already collect high‐quality, standardized clinical data, making them ideal platforms for linking molecular profiles with well‐characterized phenotypes. Even in large late‐stage trials, omics studies could be feasible as smaller sub‐studies to yield additional valuable insights into biological mechanisms and biomarkers.

In parallel, new technologies are enhancing the resolution at which immune responses can be studied. Long‐read sequencing, for instance, facilitates isoform‐level transcript analysis and the detection of complex splicing events [91]. Equally important are human‐relevant experimental systems for functional validation. Organoids, organ‐on‐chip platforms, and ex vivo tissue explants now offer physiologically relevant models for probing immune responses in specific contexts, while addressing ethical concerns associated with traditional animal models [92].

Artificial intelligence (AI) is poised to become a major enabler of systems immunology. AI methods, including machine learning algorithms, are well‐suited to learn complex patterns from large datasets and to apply learned representations to new data. In immunology, such approaches are already commonly used for a variety of tasks, such as automatic cell type annotation in single‐cell datasets [93, 94], predictions of protein–protein interactions [95, 96], and integration of multi‐omics measurements [97, 98]. Given the scale and complexity of data generated by modern omics technologies, AI approaches are particularly well positioned to characterize and explain human immune variability [98]. Foundation models, for example, enable the aggregation of data from multiple modalities and can thereby provide integrated representations of an individual's immune state based on diverse molecular and clinical inputs [99].

An important concept in this context is that of immune set points, which describe individual‐specific immune states shaped by genetic background, the environment, and other factors [98]. These set points can be inferred from multivariate biomarker profiles and used to predict outcomes such as vaccine responses or to detect subclinical manifestations of disease. In line with this concept, Molè et al. [100] identified baseline set points in vaccine high responders that resembled innate immune signatures typically induced by vaccine adjuvants, providing potential insights for adjuvant research. Understanding how immune set points are established and modulated may offer promising avenues for future research across immune‐targeted medical interventions as well as immune‐mediated diseases.

Beyond data analysis, AI may also influence the research process itself in systems immunology. A recent study evaluated the performance of large language models (LLMs) in immunological research tasks and found that these models can effectively retrieve and summarize relevant literature, propose plausible mechanistic explanations, and suggest experimental approaches when prompted with research case studies [101]. However, the study also highlighted that LLMs primarily recombined existing information and did not generate genuinely novel hypotheses or original experimental designs. Whether emerging approaches, such as having different AI agents operate in specialized roles (e.g., immunologist, structural biologist, bioinformatician, etc.) and interact with one another, can enable more creative and innovative scientific reasoning remains an open question [102].

Despite their promise, several limitations must be considered when applying AI in systems immunology, although ongoing methodological developments aim to address these challenges. Many AI models require large datasets, whereas immunological studies are often still relatively small. The COMET framework, for example, aims to improve predictive performance in small omics studies by first pretraining models on large electronic health care datasets (including a subset for which omics data is available) and transferring learned representations to these small omics subgroups [103]. Combining data from various studies represents another strategy to increase sample size; however, sensitive molecular or clinical data often restrict data sharing due to privacy concerns and regulatory constraints. Here, decentralized swarm learning could enable the integration and joint analysis of data from multiple sources while keeping data locally and preserving privacy [104]. Finally, despite the often strong predictive power of AI models, biological interpretability may be limited depending on the model used, and causal relationships can generally not be inferred. Examining internal reasoning processes of AI models may provide first hints about relationships between variables, which can subsequently be tested in targeted experimental studies [99].

Together, these developments are transforming systems immunology into a powerful framework for decoding human immunity and advancing precision medicine.

## 2 Concluding Remarks

Systems immunology offers an unprecedented opportunity to study the human immune system in all its complexity. Driven by high‐dimensional technologies, large‐scale human cohorts, and advanced analytical tools, the field is redefining how we understand and approach immune‐mediated diseases. However, realizing its full potential will require careful attention to study design, data quality, and analytical rigor. As multimodal datasets and integrative methods become increasingly accessible, the continued convergence of biology, computational science, and real‐world data holds great promise for profoundly advancing our understanding of human immunity.

## Author Contributions

L.R. and R.F. conceived and wrote the manuscript.

## Conflicts of Interest

The authors declare no conflicts of interest.

## Peer Review

The peer review history for this article is available at https://publons.com/publon/10.1002/eji.70164.
